# Responding to Risk: Regulation or Prohibition? New Zealand Media Reporting of Thoroughbred Jumps Racing 2016–2018

**DOI:** 10.3390/ani9050276

**Published:** 2019-05-24

**Authors:** Kylie A. Legg, Mary Breheny, Erica K. Gee, Chris W. Rogers

**Affiliations:** 1 School of Veterinary Science, Massey University, Private Bag 11 222, Palmerston North 4410, New Zealand; K.Legg@massey.ac.nz (K.L.); E.K.Gee@massey.ac.nz (E.K.G.); 2 School of Health Science, Massey University, Private Bag 11 222, Palmerston North 4410, New Zealand; M.R.Breheny@massey.ac.nz

**Keywords:** thoroughbred, racing, jumps racing, welfare, media framing, print and social media, public debate, social license

## Abstract

**Simple Summary:**

The acceptability of jumps racing has been debated in the media due to the high risk of horse fatality. Investigation of jumps racing articles in New Zealand for the 2016/2017 and 2017/2018 seasons demonstrated that this debate was vocal but localised and short-lived. Using rhetorical analysis, two different argumentative positions were identified, linked by a common acceptance of the inherent risks posed by jumps racing. Proponents of jumps racing advocated acceptance and minimisation of these inherent risks. This position was supported by emphasising the ‘wealth of expertise and care within the racing industry’ and was contrasted to the ‘naïve opinions’ characterising those who opposed jumps racing. Opponents of jumps racing personified the horse as a rights-bearing individual, resulting in the inherent risks becoming an unacceptable danger perpetrated by an exploitative racing industry for economic and personal gains. These different lines of reasoning provided a basis for arguments for and against the continuation of jumps racing in New Zealand.

**Abstract:**

Jumps racing involves a higher risk of accident and fatality than flat racing. The wide accessibility of media, combined with alternate views regarding the place of animals in society, raises the question of the acceptability of the continuation of jumps racing. Racing data and media articles from Newztext and Google news search were collected for the 2016/2017 and 2017/2018 jumps racing seasons, during which the fatality rate was 5.8 per 1000 starters. Jumps racing articles comprised 3.4% of all race reporting, and the volume of discussion about jumps racing was minimal (2.9% of jumps race articles related to the continuation of jumps racing), short-lived and related to horse fatalities. Articles were categorised and analysed using rhetorical analysis to determine the main arguments. The inherent risk posed by jumps racing to the horse formed a basis for two argumentative positions. Proponents of jumps racing argued that risks were reasonable, with risk minimisation measures best determined by expertise and care from within the racing industry, labelling opponents as naïve extremists. Opponents of jumps racing used anthropomorphism of the horse to argue that any risk was unacceptable and jumps racing should be banned. Horses were attributed with rights, and from this perspective, the racing industry exploited horses for entertainment. These two different arguments were used to shape claims for and against the continuation of jumps racing, allowing both to be built upon a shared acceptance of inherent risk.

## 1. Introduction

Jumps racing is a form of thoroughbred horse racing that includes hurdling and steeplechasing. It occurs in 18 countries on four continents and traditionally is of symbolic, cultural and economic significance [1]. Horse racing was introduced to New Zealand by English settlers in the early 1800s. By the 1880s, New Zealand had more racecourses and racing clubs per capita than anywhere in the world [2]. Jumps races comprised 4% of all races run in NZ between the years 2005–2011, with a median number of nine starters per race [3]. Jumps race distances are greater than flat races, ranging from 2900–4000 m (IQR) with approx. 25 jumps compared to 1200–1670 m (IQR) for flat races [3]. There are no accurate figures of average attendances at race meets, but it has been estimated that 361,471 people attended thoroughbred race meetings throughout New Zealand in 2016–2017 [4]. However, race day attendance does not necessarily reflect interest, due to widespread media reporting of races and off-course gambling opportunities [2].

Although important symbolically, the economic impact of jumps racing is more limited than flat racing. Jumps racing occurs only in the winter months, with fewer races at fewer locations and with limited prize purses compared to flat racing [2,3]. There is a concern that jumps racing is a dangerous discipline that can be particularly harmful to the horse due to the long distances raced, number of jumps and speed of racing. Due to these factors, jumps racing is controversial primarily because, compared to flat racing, it involves a higher risk of accident or fatality to both horse and rider [5,6]. Worldwide fatality rates for jumps racing range from 3.9–8.3 per 1000 starts in contrast to that for flat racing of 0.44–1.9 per 1000 starts [7,8,9,10,11,12]. Fatalities in jumps racing continue to occur, and with the wide accessibility of the media, graphic images and videos of falls reach a wide audience, not just racing enthusiasts and those present on race day. This has led to debate in the media prompted by animal welfare and rights groups and individuals concerned about the acceptability of jumps racing as a sport [2,11,13]. As a particular example of a human–animal activity, jumps racing is viewed variously as exciting, archaic or barbaric [1]. The limited economic viability together with concerns for horse and jockey safety has contributed to the marginal and contested nature of jumps racing.

Changing social values and new technologies have contributed to increasing media attention and public debate about the acceptable use of animals in sport [14]. The acceptable use of animals in sport has been discussed in terms of social license [15]. Social license is a relatively new concept, which considers the role of the community or wider society in sanctioning or censuring activities, such as equine sport. It is rooted in the beliefs, perceptions and opinions held by the local population and as such is intangible, dynamic and transient, reflecting the changing attitudes of society. Debates about jumps racing around the world have been played out via mass media and have relevance in the ongoing cultural renegotiation of the meanings, norms and governance of human–animal relationships in modern societies [11]. Two key factors have influenced changes in the way racehorses are understood by most people: The increased presence of animal rights/protection groups and communication technology changing the way horse racing and associated welfare issues are presented and debated in the media [14]. Media portrayal of issues involving animals can influence public perceptions, which may impact on government policy or other actions in relation to animals [2]. The recent debate (2018) in the UK parliament concerning racehorse fatalities and the need for an independent authority to oversee horse welfare is a prime example of animal rights groups influencing media reporting, leading to political debate [16].

Mainstream media accounts are not objective; content is influenced by a number of factors, including ‘newsworthiness’, influence of politicians or media owners and the personal views of the authors [17]. Jumps racing in Australia has been hotly debated in the media since 2008, due to an unusually high fatality rate and a comprehensive jumps racing review [13]. Accidents and fatalities in jumps racing are highly public and often involve spectacular falls, providing activist groups with graphic photographs which can be used strategically to raise public awareness and shape public opinion. In particular, images can be used to fuel criticism of racing and of the racing industry’s approach to equine welfare [13]. Increased visibility is used to promote concern regarding welfare outcomes, resulting in improved protective legislation for these high profile animals [11]. Arguably, public concern about horse welfare has contributed to the decline of jumps racing in Australia, although economic factors such as the smaller scale of jumps racing compared to flat racing have also been significant [1]. Since 2008, industry- and government-led reviews in response to media campaigns in Australia have resulted in modified racing regulations aimed at increasing horse and rider safety. However, despite temporary suspensions of the sport, jumps racing and the associated debate in Australia is ongoing.

There are common arguments employed by both sides in the jumps racing debate. Advocates of jumps racing argue that horses love to race and jump, that jumps racing extends a horse’s career and that many of the horses would be slaughtered if not for jumps racing. Opponents argue that horses have evolved to avoid rather than jump obstacles, the higher risk of injury or death of jumps racing horses is an unacceptable focus for human entertainment and that this risk is not an acceptable alternative to the slaughterhouse [11]. These arguments form part of the contested nature of jumps racing in the media. Beyond these simplistic arguments, there are also subtle ways that the claims are made and undermined that are important in justifying or resisting the continuation of jumps racing.

The aim of this study was to determine the quantity of media reporting of jumps racing in New Zealand and its’ association with horse casualties. Secondly, the study aimed to identify the key arguments used in the New Zealand jumps racing debate.

## 2. Materials and Methods

The extent of jumps racing in New Zealand and incidence of casualties therein were determined by analysing racing data from New Zealand Thoroughbred Racing Inc (NZTR), the governing body for horse racing in New Zealand, covering two racing seasons, 2016/2017–2017/2018.

Articles from New Zealand newspapers accessible online and written between 1 May 2016 and 31 August 2018 (2 racing seasons) were collected and analysed. A search was conducted using two main search engines: The Newztext database (including national and regional newspapers) and www.google.co.nz news section. This search used the keywords ‘horse rac*’ OR ‘horserac*’ OR ‘race*’ AND ‘thoroughbred’ OR ‘hors*’. The * denotes any ending. Following this, the articles were limited to those pertaining to jumps racing, by using the keywords ‘jump*’ OR ‘hurdl*’ OR ‘steeplechas*’.

Original articles were printed in newspapers or their associated online sites with up to 6 duplicates of newsworthy articles in different newspapers. In 2014, the average issue readership of the New Zealand Herald was 470,000 (13% of the population), and the average issue readership of all regional daily newspapers was 835,000 (23% of the population) [18]. The New Zealand Herald online site was visited by 44% of New Zealanders, overall attracting 1.7 million visitors a month or 64% of the total online audience in 2011 [19]. In the same year, online media news site www.stuff.co.nz was visited monthly by 52% of New Zealanders over the age of 18 and www.scoop.co.nz by 11.2% [20]. This indicated that online media reaches a greater number of people than print media. However, most print media were also able to be accessed via newspaper online sites.

The identified articles were saved to an excel file with title and media type/section identified. Duplicate articles were recorded but only one original copy kept. The articles were categorised into neutral–positive (articles reporting generally on jumps racing), neutral (articles portraying both positive and negative aspects of jumps racing), positive (any article that specifically mentioned jumps racing in a positive light) and negative (articles clearly opposed to jumps racing). All articles fitted simply into these codes.

The positive, negative and neutral articles were saved into NVivo (NVivo 12 Pro) and examined using rhetorical analysis. The key feature of rhetorical analysis is argument, which is a fundamental part of peoples’ everyday lives. This analytic approach focuses on how individuals formulate arguments and construct self, others, objects and occurrences as well as anticipate and counter other argumentative positions [21]. Rhetorical analysis examines the ways in which argument is constructed through text or talk to influence an audience towards accepting particular versions of reality or courses of action [22]. Thus, the main objects of analysis were the arguments (or lines of reasoning). Ideas and values are the foundation for everyday argument, and as such, the arguments only have meaning in context and in relation to alternative arguments.

The initial stages of analysis involved reading and re-reading the data and becoming familiar with the contents. The articles were examined for overall meaning and tone before identifying common themes or arguments. Instances of similar reasoning were grouped together into descriptively labelled themes. All authors discussed the coding and agreed on the assignment of data to thematic categories. Prominent argumentative themes were examined, and the historical, rhetorical and situational context of the articles considered throughout. The data were examined for rhetorical constructions. The function of the text to achieve a purpose, construct a version of the world, or position people in a certain way was considered before grouping common arguments together to form nodes. To represent these different argumentative positions, the data were analysed in terms of the protagonists of these claims (see Tables 3 and 4). Extracts which best represented each of the prominent themes were used as illustrative examples. All authors approved the final analysis.

## 3. Results

### 3.1. Horse Racing Casualties

There were 293 jumps races during the 2016–2018 jumps racing seasons. These races comprised 4.9% of the total number of horse races in New Zealand during that period. During these races, there were 15 horse fatalities, resulting in a fatality rate of 5.8 (3.4–9.3, 95% CI) per 1000 starters.

**Figure 1:**
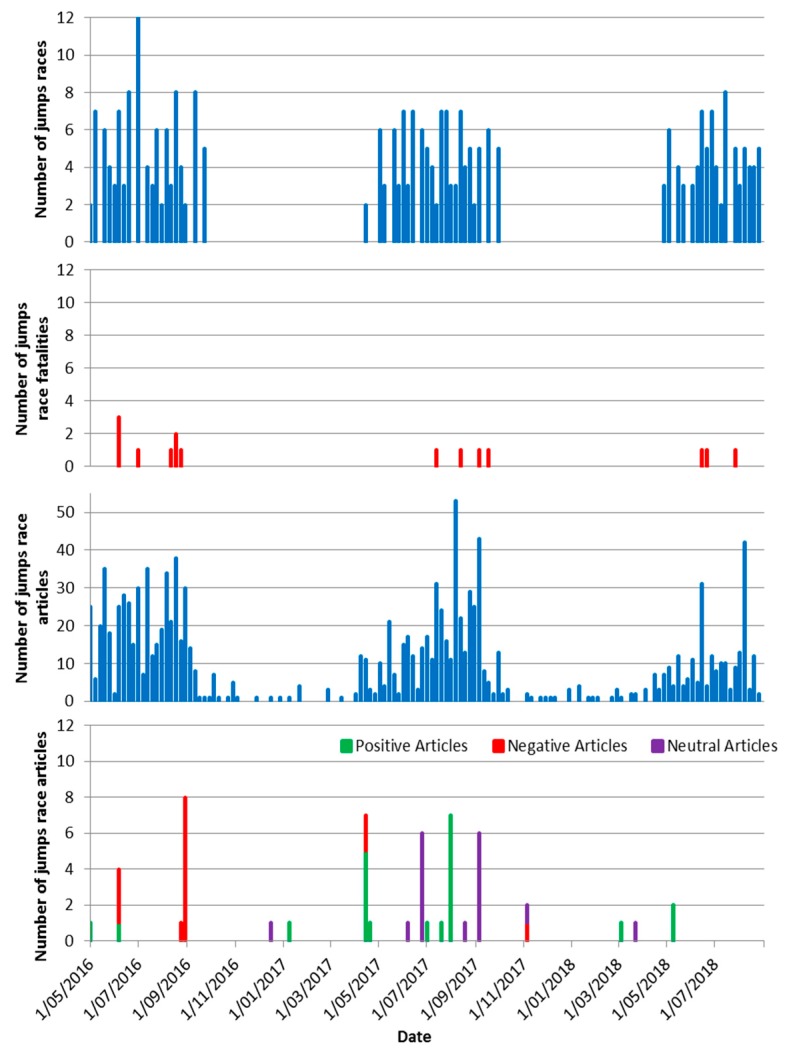
Number of jumps races (1st graph), number of fatalities (2nd graph), number of articles on jumps racings (3rd graph) and tone of articles covering jumps racing in New Zealand (bottom graph) from May 2016–August 2018.

Figure 1 shows the incidence of jumps races, jumps race reporting and jumps race casualties over the two-year period from May 2016 to August 2018. The number of articles reporting jumps racing varied accordingly with the races during the jumps season. Positive articles were distributed throughout the jumps season, and negative articles followed horse fatalities, either in New Zealand or Australia.

During the period from 1 May 2016–31 August 2018, 36,204 articles on horse racing were identified in New Zealand. Of these articles, 1228 articles (3.4%) pertained to jumps racing. Of these jumps racing articles, 96% were classified as neutral–positive (reporting on horse form, race results, events and preparation). There were seven neutral articles (with ten duplicates), representing 1.4% of all jumps racing articles; these included articles that were neither race reports nor relevant to the jumps racing debate. Horses falling during a jumps race were mentioned in 9% (60/683) of articles. The sports or racing sections of the news source contained 87% of jumps racing articles.

Ten distinct articles (1.5% of all jumps racing articles) were classified as positive towards jumps racing (with 11 duplicates). Print media/newspaper sites published nine of these articles, with two available on the online media site stuff.co.nz. There were eight negative articles (with seven duplicates) pertaining to jumps racing, representing 1.2% of all jumps racing articles. Three of these articles were found in print media and five on online media sites (three on scoop.co.nz, two on stuff.co.nz). In total, articles reporting clear views supporting or opposing jumps racing represented 2.9% of all jumps race reporting. A recent horse casualty (either in New Zealand or Australia) at a jumps race was mentioned in 8/8 negative articles and 3/10 positive articles.

Flexible rhetoric strategies were used to present the arguments advocating for, or condemning, jumps racing. The arguments focused on four main points: The inherent risk of the sport, the role of jumps racing in society, the effect of race day accidents, and possible solutions to decrease horse fatalities. The arguments supporting and opposing jumps racing are summarised in Table 1 and Table 2. Justification given for each argument’s line of reasoning were made by attributing different values to the three main protagonists in the jumps racing debate: The proponents of and opposition to jumps racing, and the horses themselves. These attributes are summarised by illustrative quotes in Table 3 and Table 4.

### 3.2. Supporters of Jumps Racing Arguments

Proponents of jumps racing used the well-known and accepted risks of jumps racing as a basis for their ‘accidents happen’ approach to fatal accidents. The traditional and exciting aspects of the sport were emphasised, and the solution proposed was that of accepting injury as an animal welfare issue with ongoing reviews to increase safety. The expertise of the racing industry, alongside a purported ‘love of the horse’ was touted as both reason and justification for understanding the horses’ wants and needs.

#### 3.2.1. Inherent Risk

Proponents of jumps racing were fatalistic about the widely accepted risks of jumps racing. The risks were simply stated, and these risks were compared with the general risks of other equestrian activities and sports. This normalised the risks, equating them with everyday activities, as Singh [28] elucidates:

*“We have camaraderie because we all know we could be falling over any day and we go out with high hopes some days and not realise that.”*

This argument appeals to the national identity of New Zealand as a tough, thrill-seeking nation with an acceptable degree of risk for the pursuit of entertainment. The risks are well known and accepted by the racing community and supporters as the norm. Intimate knowledge and acceptance of these risks provided a foundation for belittling the claims of jumps racing opposition. Arguments against the common view were pre-emptively dismissed:

“Jumps racing is a misunderstood discipline….”

[32]

This dichotomy sets up the racing industry and their supporters as well-informed rational thinkers, both aware and accepting of the inherent risk in the sport. At the same time, opponents to this way of thinking were styled as radical and ignorant.

#### 3.2.2. Jumps Racing Role in Society

The underlying narrative from mainstream media reporting of jumps racing was that of a ‘crowd pleasing’ [25] and exciting sport which brings the community together. References to ‘thundering hooves’ [24] were suggestive of horses running in the wild as well as evoking a sense of excitement and activity. 

Supporters of jumps racing emphasised the traditional and social value of the racing industry, providing jobs and entertainment. The racing industry was portrayed as a caring industry with strong community ties, for example:

*“It’s got a very big unity focus in the sense that people come together in our country to enjoy race track meetings”*.

[25]

This harks back to the foundations of New Zealand society—based on camaraderie, toughness and tradition. The implication was that racing is an integral part of society and as such should be protected. Thus, the assumption was that the views of the racing community were shared by a large, unified group of New Zealanders. This sense of community and unity was used to portray the racing industry and supporters as knowledgeable, experienced and informed, and to set them against those who opposed jumps racing and were styled as radical, naïve and uneducated:

*“The extremists may have meant well, but, through their ignorance, more horses than ever were in demise”*.

[34]

These naïve responses to jumps racing were viewed as more damaging than the racing itself. This claim refers to (unsuccessful) measures taken to increase horse safety in jumps racing in Australia, in response to protests from animal rights groups. Attributing jumps racing opponents as well-meaning but ignorant adds weight to the claims of experience within the racing industry as able to know what is best for the horse. This account includes the reader in the assumed majority group of people supporting jumps racing whilst disparaging anyone who opposed this view as naïve and misinformed, though potentially well-meaning.

#### 3.2.3. Race Day Accidents—Death/Injury

Fatal accidents were treated as tragic in articles supporting jumps racing but presented as unusual aberrations in an otherwise safe practice. The acceptance and elucidation of the inherent risks of the sport provided both a basis and excuse for their reaction to fatal accidents. The social acceptance and trivialisation of the inherent risk in the sport was summed up by Hill [35]:

“But statistically where do you draw the line? It’s not in anyone’s vision that we want to see horses that have fatal injuries, but we sit very well compared to all forms of sport.”

This suggests that risk falls on a continuum from no risk to fatal injuries. Whilst acknowledging that fatal injuries are clearly an outcome that is not in racing proponents’ vision for the sport, drawing the line before this is presented as an arbitrary assessment of acceptable risk according to what is deemed appropriate for society. The comparison is drawn with all forms of sport to demonstrate that risk is pervasive. 

Nevertheless, individual deaths were not marginalised; instead, their psychological effect on the industry was emphasised:

*“The death of any horse is a tragedy and the people of the industry are devastated”*.

[23]

Through this emphasis of an emotional reaction to every death, claims from industry professionals of deep attachment to their horses were strengthened. This theme was used throughout as a pre-emptive strategy against jumps racing opposition claims of an unfeeling and exploitative industry and to emphasise that though the risks were accepted, they were not taken lightly.

#### 3.2.4. Solutions to Reduce Horse Fatalities

Through the acceptance of the inherent risk within the sport, the solutions to minimising fatal accidents within the industry were cast as an animal welfare issue. As such, reviews were suggested to identify risk minimisation strategies according to welfare guidelines. Indeed, these measures were touted as ongoing investigations into the improvement of the sport as expressed by Walters [23]:

“NZTR also invested more than $1 million in racing and training infrastructure improvements in the past financial year, to maximise equine safety and welfare.”

The basis for the arguments supporting jumps racing rested on the acknowledgement of the wealth of expertise and care of the racing industry, meaning that they were able to make rational judgements in the assessment and mitigation of the acceptable risks of the sport to the horses involved.

#### 3.2.5. Attributes of Supporters, Opposition and Horses Involved in Jumps Racing

Those involved in jumps racing were represented as inevitably bound to the fate of their horses. This was used throughout the articles to pre-empt any suggestions that trainers, jockeys or the industry as a whole was unfeeling and exploitative. This claim of emotional attachment was used to undermine the claims that racing proponents were cruel and unfeeling; it suggests that cruelty and exploitation cannot co-exist with love. The proximity to the horse was used to demonstrate both expertise and attachment, which played an important role in who could ‘speak’ for the horse. This allowed the opinions of those outside the industry regarding equine welfare to be dismissed as irrelevant:

*“People like me, who have had horses all their lives, love horses more than any animal rights person.”*

[23]

This focus on knowledge and expertise also played out in terms of the ways that the horses themselves were described. The owners, trainers and jockeys were described as knowing what was best for the horse, whereas the horses themselves were characterised as spoiled children, requiring care and attention. Blame for accidents on the racetrack were placed on the horses themselves (when they made a mistake):

*“Early leader Nells Belle had ditched jockey Shaun Fannin off her back to cross the line in race seven here on Saturday.”*

[32]

However, jumps racing success stories highlighted the personal lives of the trainers and jockeys, for example:

*“Last winter the Whanganui based trainer won four of the nine jumps races at the carnival and nine of the 32 races overall.”*

[31]

In articles supporting jumps racing, the horses were secondary to the human stories of devotion and success. This centralising of the human participants in the racing industry supported the arguments that industry experts are best placed to decide what is best for the horses.

Together, these arguments were used to build and strengthen an argument for the continuation of jumps racing. This began with the accepted risk that ‘accidents happen’ and are inevitable. Deaths were constructed within the narrative as singular aberrations in an otherwise regulated and controlled industry. The justification for ‘knowing best’ and doing the right thing was experience with horses and proximity to and love of the horses; the horses were characterised as disorderly children needing management.

*“We love our horses. That’s why we’re in it. We’d be in it whether there was prizemoney or no prizemoney”*

[36]

### 3.3. Opposition to Jumps Racing Arguments

The opposition to jumps racing argument also began with the view that jumps racing was inherently risky and as such, fatal accidents were inevitable. From this shared beginning, opponents concluded that the only solution was to ban the practice. Opposition to jumps racing arguments were emotive, establishing their argument through personification of the horse as entities with human feelings and basic (human) rights. Jumps racing was portrayed as an exploitative and unnatural activity for the horse, and therefore, it was deemed unacceptable to force these known risks upon a rights-bearing individual. Enlightened, forward thinking and empathetic views were touted as reasons and justification for understanding the horses’ wants and needs alongside the assumption that the horses were unwilling participants.

#### 3.3.1. Inherent Risk

The risks inherent to jumps racing were deemed obvious and clear to the audience by jumps racing opponents. They were explicitly stated with the added claim that the horses were ‘forced’ to participate. This unwillingness to participate was used to imply that this in itself added to the risk of the already risky sport. For example:

*“SAFE condemns jumps racing because of the inherent danger to the horses who are pushed to jump fences at speed, resulting in injury and death.”*

[30]

In this way, the danger of the sport was viewed as in part a result of the horses’ unwillingness to participate. Potential and actual accidents were vividly described to elicit sympathy from the audience. Taken further, this assumption of being forced into a potentially deadly situation linked horses in jumps racing to that of innocent victims in a war, with its associated lethal connotations. This was achieved by personifying the horse, attributing them with human values, actions and feelings and implying they had foreknowledge of potential harm. This allowed the reader to relate to the horses’ experiences as if they approximated human experience and obliquely attributed the same rights to the horse as that of a human, for example:

*“..the animals themselves are running scared, knowing they are in danger of injury and death and powerless to do anything about it.”*

[33]

This encouraged the audience to compare themselves with the horses portrayed, thus highlighting what exactly is perceived to be an acceptable risk and to whom or what. By personifying the horse, people were able to project their own feelings and responses on the horse. This enabled the audience to become experts on the situation of jumps racing and thus allowed the ‘voice’ of the horse to be universally interpreted. No longer is knowledge of horse welfare required to speak knowledgeably about or question the racing industry.

#### 3.3.2. Jumps Racing Role in Society

Jumps racing was depicted as a flagrantly exploitative activity, resulting in only personal gain to the industry’s supporters. The defenceless horse was subjected to the whims of humans, epitomised by the racing industry. The horse was portrayed as central to the story with their welfare and rights paramount by using rhetorical constructs and imagery linked with human atrocities, thus evoking a sense of kinship with the horse, for example:

*“Injuries suffered when horses fall or crash into jumps or barriers can be horrific. Animals that are no longer deemed able to race profitably are discarded, often ending up at the slaughterhouse.”*

[30]

This idea emphasised the needless and exploitative nature of jumps racing, likening horses to disposable objects used for human entertainment and profit. The ‘artificially-created’ [33] nature of racing was emphasised in contrast to the utopian view of a horse in the wild, which was previously evoked by the racing industry’s reference to ‘thundering hooves’.

Acceptance of the cruelty of jumps racing was linked to outdated beliefs as well as apathy and disinterest from the masses. It was implied that those not against the industry were part of the soundless mass whose silence enabled the horses’ suffering. Horse racing was described as a vestige of past days, where people ‘used them [horses] in battle’ [33] and now subject them to similar ‘horrors’ for entertainment. The implication was that the racing industry and those that support them were behind the times and needed to advance their views on animal rights and welfare in line with the advanced reasoning of today, for example:

*“Racing horses over jumps poses an unacceptable risk to both horses and riders. It’s cruel, it’s barbaric and it doesn’t belong in the 21st Century.”*

[36]

Using this argumentative frame, a superior moral position was attributed to the minority group opposing jumps racing and provided the grounds for personal heroic action. This position of the minority of enlightened and concerned activists was enlisted in a call to action:

*“If not us, who? If not now, when?”*

[33]

#### 3.3.3. Race Day Accidents—Death/Injury

Fatal accidents were treated as inevitable consequences of the inherent risk of jumps racing. Deaths were counted as ongoing casualties in a war, encouraging the audience to identify and sympathise with the plight of the horse. Used in this way, the ever rising ‘death toll’ evoked imagery of needless deaths and provided powerful justification for the defence of the rights of the horse. Ryan [26] elucidates this:

*“The death toll in New Zealand jumps racing has risen with three horses dying in three weeks - the total for the jumps season is seven.”*

The numbers of horses euthanised within a given time period of racing was frequently mentioned, drawing attention to recent fatalities and ignoring long-term trends with little to no context of the number of horses involved. This matched the justification that they provided for concern, the humanising of the horse as an individual who experiences fear and pain and should be accorded rights. From this perspective, the rate of injury is less important than that it occurs at all. Any injury or death of an unwilling participant is therefore unacceptable.

#### 3.3.4. Solutions to Reduce Horse Fatalities

The personification of the horse and comparison with human attributes led to the issue becoming one of animal rights. Coupled with the identification of the inevitable inherent risk in jumps racing, there was only one logical solution:

*“Jumps racing is impossible to make safe, as by its very nature there is a constant risk to the horse…..The only solution is to ban jumps racing.”*

[29]

Those that opposed jumps racing were clear and unwavering on their solution to jumps racing death and accidents. This solution was based on the premise that horses are rights bearers, and they used personification to allow the audience (and themselves) to have the authority to ‘speak’ for the horse by direct comparisons with human rights. As rights bearers, their rights logically should not be abused.

#### 3.3.5. Attributes of Supporters, Opposition and Horses

Opponents of jumps racing set themselves up as a forward-thinking minority—fighting for a higher moral good against the exploitative racing industry and their supporters. Their argument was supported by comparison with other countries worldwide; in particular the Australian jumps racing debate:

*“New South Wales has already banned jumps racing. SAFE says that New Zealand needs to do the same.”*

[30]

The implication was that Australia (and the rest of the world) are progressive, forward thinkers, with New Zealand lagging behind with its acceptance of ‘the high rate of injury and death’ [27] in jumps racing. In addition, comparison with Australia effectively increased the population of jumps horses and races, thus increasing the number of accidents able to be commented upon.

Horses became the main focus and protagonists of the articles opposing jumps racing. These articles were written in every case after a specific fatal accident. Horses were accorded more significance in the articles opposing jumps racing than the articles supporting jumps racing. They were anthropomorphised in this argument as victims of human exploitation: ‘suffering’ accidents after being ‘forced’ to run in ‘terror’ and eventually adding to the ‘death toll’. Other industry practices such as the fate of unsuccessful racehorses and overbreeding, as well as the use of the whip, were used to condemn the entire industry as cruel.

Claims of ‘love of the horse’ from the racehorse industry were dismissed by jumps racing opposition. It was implied that the reputed industry members’ attachment to their horses was that of an object and was conditional and proportional to its return on investment. It was claimed that profit was more important:

*“The lives and welfare of the horses are what is really at stake in jumps racing: an industry where profit and prestige out-weigh the safety of the animals.”*

[30]

However, ‘love’ was not claimed by the opposition to jumps racing. Instead, the presence of inherent risk in the sport and implied rights of the horse were enough to condemn the practice.

## 4. Discussion

The findings in the present paper indicated that the visibility of jumps racing in New Zealand media was low over the 2016/2017–2017/2018 period and was not a prominent issue in mainstream New Zealand media. The volume of discussion of the debate over the two-year study period was minimal in comparison to the volume of jumps race reporting, or even race reporting overall.

The quantity of jumps race reporting (3.4% of total race reporting) reflected the quantity of jumps races as a percentage of all thoroughbred racing in New Zealand (4.9%). This indicated that the volume of discussion of jumps racing in the media followed simple race coverage throughout the racing season, with only 2.9% of jumps racing articles specifically addressing the role of jumps racing in society. The print media were generally very supportive of jumps racing, and the majority of negative articles were found in online media. However, online readerships match those of the print media outlets and associated online sites, so both positive and negative articles were likely to reach similar numbers of people.

There were no noticeable spikes in reporting after a race casualty, indicating that these events did not receive widespread attention by the New Zealand media. However, the timing of the reporting for the negative articles was significant—these were published in every case following an incident in a jumps race. These incidents did not always occur in New Zealand but included Australian jumps racing fatalities, thus capitalising on a larger pool of horses likely to have an accident. This gave the opponents of jumps racing highly visible material to engender sympathy and support for their arguments. In addition, comparisons were drawn with the debate on Australian jumps racing by both those supporting and opposing jumps racing, using evidence and incidents from Australia to add weight to their arguments. However, reports quickly died away within a week of the first published article, indicating that there was minimal discussion or interest surrounding the event. The major area of contention between advocates and opponents was the justification of jumps racing—whether it was an accepted and integral part of society or a forced and cruel sport.

### Article Analysis

How jumps racing events were depicted in the media was significant given that most readers were unlikely to have been eyewitnesses to events. Media outlets can be considered ‘gatekeepers’ of information as they are able to select the information to be published and how it is portrayed [37]. Thus, the media are important to the particular construction and perpetuation of morality and therefore influence the social license of the industry to operate [13]. Articles in support of jumps racing framed the inherent risk of the sport as acceptable and relatively inconsequential, similar to attitudes found in other sporting fields [38]. Arguments against this view were depicted as naïve. The opposing articles presented animal rights and welfare as the main issue [39] and maintained that our moral responsibility extends to other forms of life than human; thus, inflicting risks upon animals was not acceptable. These accounts are strategically crafted to highlight particular arguments and to undermine potential alternatives.

The construction of the horse and their relationship with humans is complex, with humans representing the horse in media debates [13]. This challenged the currently accepted social license of the racing industry to operate, by using the perceived viewpoints of horses to influence public opinion. Both supporters and opponents of jumps racing arguments shared the same original premise—that jumps racing posed an inherent risk to the horse. However, the arguments and conclusions drawn as to why and how this risk was acceptable and the solutions to mitigate this risk diverged. This divergence stemmed from who could ‘speak’ on behalf of the horse and the interpretation of what they would choose if allowed a choice. Central to this construction was the unequal power relationship between humans and horses, with humans having ultimate control over the horses’ lives [14].

The proponents of jump racing arguments were built on human participants as the central characters in horse racing. Proponents suggested that industry and equine expertise was required to understand what is best for the horse. The emphasis was on the relationship between horse and trainer/jockey as caring and supportive. The horse was described as both a ‘loved’ and working animal, highlighting that it is possible for emotionality and instrumentality to co-exist in equine industries [40,41]. This affection professed by industry members was implicitly given as evidence of good treatment for the horses in their care and implied that the horses would not then be exposed to an unacceptable risk. Very little recognition was given to the equine–human power relationship, with horses requiring care, management and ‘stimulation’ [32] from their human caretakers. This emphasis on the proximity and expertise as the foundation for understanding jumps racing encouraged the reader to accept the opinion of the racing industry.

Oppositional arguments were built on horses as the central characters. They suggested that industry experts were bound up in the exploitation of horses and so could not provide an unbiased opinion. Instead, they proposed those at a distance from the industry were best placed to provide a voice for the horse. By attributing the horse with human rights, thoughts and feelings, equine experience was negated, and anyone could participate knowledgeably in the argument regarding jumps racing. The fates of specific horses involved in catastrophic accidents were used as a trope for all horses involved in jumps racing. Thus, equine welfare and rights became identifiable with personal welfare and rights and formed the basis for the argument that the risk to all horses posed by jumps racing was unacceptable. The power relationship between horses and humans was emphasised, highlighting the transient nature of the industry ‘discarding’ [33] horses when they were no longer useful and the purported ‘love’ conditional on the horses’ financial return. The negative articles were explicit in condemning this power relationship as exploitative.

There was no common ground between the supporters and opponents of jumps racing, with opponents taking the unyielding view that the sport should be banned. The supporters of jumps racing acknowledged the risks of the sport, deeming them acceptable, and compared them to other accepted horse activities which also have inherent risk. Proponents used this to advocate for adherence to industry safety regulations. However, the narrative from those opposed to jumps racing was that of exploitation of horses for human profit and entertainment. The life of each horse was made significant and likened to human atrocities. This framing of the importance of individual animals, illustrated with graphic images and videos, enabled the media to publicise stories of relatively rare injuries and fatalities to a much larger audience than those present at horse races or involved in the racing industry.

The importance of images in the articles cannot be underestimated. These included photos and YouTube video footage of jumps racing accidents posted by jumps race opposition on media websites. The graphic details of the accidents were thus able to be seen and experienced by many. Out of context of the totality of jumps racing events, these incidents could be framed as the usual and common outcome of jumps racing. By contrast, articles supporting jumps racing printed pictures of horses in full health and flight or emphasised the human aspect of a loving relationship between horse and trainer or jockey. These images provided support for the argument that experts within the industry know what is best for the horse. Analysis of images and video was beyond the scope of this study, but inclusion of these as data sources would be a valuable avenue for future research on ways that jumps racing is represented in the media.

Understanding the lines of reasoning behind each argumentative position in the jumps racing debate can help to reveal how these arguments can influence and shape public opinion. At present, the arguments used prevented any movement towards shared solutions to the danger of jumps racing. This recognition could open avenues for informed and knowledgeable discussion of the risks and reasoning for jumps racing between the racing industry and those who oppose it. Such discussion could result in measures being taken that are acceptable to both parties.

## 5. Conclusions

Jumps racing generally elicited little interest from the mainstream New Zealand media. Reactions to horse casualties were immediate, and articles condemning jumps racing used these events as opportunities to promote their arguments. However, little discussion was generated, and attention typically receded within a week of any incidents.

The inherent risks of jumps racing were universally accepted and formed a basis for both argumentative positions for and against jumps racing. Jumps racing supporters had the pragmatic position that the traditional benefits of the sport (for both profit and entertainment) alongside industry measures to safeguard the health and welfare of the horses justify the continuation of jumps racing. Deaths were argued to be singular aberrations in an otherwise regulated and controlled industry. Opponents to jumps racing viewed jumps racing as exploitative, posing a high and inevitable risk to the horses involved. Horses were personalised and attributed with rights. Therefore, deaths were argued to be needless, horrific and tragic. Thus, the similar reactions to a horse death in a jumps race—that of a ‘tragedy’—were given entirely different meanings when considered either as a rare and worrying occurrence inducing stricter welfare guidelines or as an inevitable outcome of a sport which should be banned.

Understanding the rhetorical constructions of such positions can reveal why particular arguments might gain power, opening the way for a more knowledgeable and informed positioning of both individuals and industry to emerge in public debates on jumps racing.

## Figures and Tables

**Table 1:** Arguments from 10 articles supportive of New Zealand jumps racing published between May 2016–August 2018.

Inherent Risk	Jumps Racing—Exciting, Traditional Sport	Death/Injury—Concerning	Solution—Increased Management
“There is always an element of risk, to both horse and rider, in all equestrian sports but NZTR (New Zealand Thoroughbred Racing) is committed to doing everything we can to mitigate those risks.” [23] “Whatever you do with horses there will always be accidents and injuries,…” [23]	“The racecourse will once again resonate with the sound of cheers and thundering hooves.” [24] “While there *[sic]* welfare and safety concerns around jumps racing, Bennett said yesterday gave an ideal snapshot of the crowd-pleasing aspect of it.” [25]	Deaths “unusual” or “worrying” “The series of unfortunate events began with Tu Meta Peta falling and fracturing his right shoulder after awkwardly jumping the first fence at the Queen’s Birthday weekend meet. The five-year-old gelding had to be euthanised.” [23] “Any fatalities are obviously disappointing and distressing for all involved.” [26]	“We’ll look at the design of obstacles, the track conditions making it more dangerous and ensuring jockeys don’t race on tired horses.” [26] “NZTR also invested more than $1 million in racing and training infrastructure improvements in the past financial year, to maximise equine safety and welfare.” [23]

**Table 2:** Arguments from eight articles opposed to New Zealand jumps racing published between May 2016–August 2018.

Inherent Risk	Jumps Racing—Exploitation	Death/Injury—Mounting Death Toll	Solution—Banning
“No one should be surprised that we’ve had yet another horse killed by this inhumane activity.” [27] “…due to the “horrific” injuries horses suffer after falling or crashing into jumps, which often lead to their deaths.” [28]	“…because of the inherent danger to the horses who are pushed to jump fences at speed, resulting in injury and death.” [29] “…an industry where profit and prestige out-weigh the safety of the animals.” [30]	“The death toll in New Zealand jumps racing continues to mount with three horses killed in three weeks, bringing the total for the jumps racing season so far to seven.” [30] “…it was only matter of time before the first horses were killed in this year’s jumps race season.” [29]	“Jumps racing is impossible to make safe, as by its very nature there is a constant risk to the horse…..The only solution is to ban jumps racing.” [29]

**Table 3:** Attributes of protagonists in 10 articles supportive of New Zealand jumps racing published between May 2016–August 2018.

Racing Supporters	Racing Opponents	Horses
Pride, expertise, commitment, care, traditional values “Trainers, jockeys, owners, strappers and many other men and women work with these horses day in and day out, putting significant time, effort and love into the care of these important animals, and the losses are felt keenly by all.” [23]	Naïve, extremists, unworldly, idealistic “Nothing stirs up the anti-racing brigade like jumps racing. But many perceptions are either exaggerated or simply untrue.” [31]	Disorderly, cared for, pampered “…riderless horses stole the thunder from the field…” [32] “At 3pm each day, she said, the animals would knock on their doors to be fed…” [32]

**Table 4:** Attributes of protagonists in eight articles opposed to New Zealand jumps racing published between May 2016–August 2018.

Racing Supporters	Racing Opponents	Horses
Exploitative, old fashioned, greedy, manipulative “People are betting, while owners and trainers are making money, and it is costing the horses their lives.” [29]	Forward thinking, moral “Racing horses over jumps poses an unacceptable risk to both horses and riders. It’s cruel, it’s barbaric and it doesn’t belong in the 21st Century.” [33]	Terrified, exploited, suffering “…the animals themselves are running scared, knowing they are in danger of injury and death and powerless to do anything about it.” [33]
